# Systematic Reviews and Meta-Analyses to Inform Recommendations for the Final Phase of Elimination and Prevention of Re-Establishment of Malaria

**DOI:** 10.4269/ajtmh.24-0151

**Published:** 2024-04-02

**Authors:** N. Regina Rabinovich, Richard W. Steketee

**Affiliations:** ^1^ Malaria Elimination Initiative, ISGlobal, Barcelona, Spain;; ^2^ ExxonMobil Scholar in Residence, Harvard TH Chan School of Public Health, Boston, Massachusetts;; ^3^ Malaria Consultant, Bethesda, Maryland

The guiding principle of Ministries of Health, the World Health Organization (WHO), funders, and implementers in global health is that policy recommendations should be based on robust scientific evidence. Populations deserve access to tools that are proven to be safe and efficacious and can be delivered to ensure that everyone in need is reached. Robustly designed primary studies and post-licensure operational studies to answer key questions defined by countries allow for tailoring implementation strategies to best accomplish public health goals.

For global health recommendations, creating such policies is far more difficult than one may expect. The WHO needs to demonstrate that the evidence resulting from different studies addresses a stringent set of considerations, including that the research questions are clear, and that the evidence includes: benefits and harms, certainty, avoidance of bias, consideration of values and preferences, financial resources required, equity, acceptability, and feasibility. Often, studies address different primary questions, are implemented in widely varying geographies and epidemiological environments, and use very different study designs. This is particularly true in the field of malaria, where the parasite, mosquito vectors, and human response to infection vary by biology, geography, and season. Unfortunately, given budgetary limitations, studies are often underpowered to answer their priority questions.

Generally, we consider systematic reviews and meta-analyses for the most robust evidence. The supplement accompanying this article provides a set of such studies. Commissioned and led by the WHO, teams from the US Centers for Disease Control and Prevention and MESA (hosted by ISGlobal) screened more than 10,400 individual papers for the systematic reviews, and ultimately identified 90 (0.86%) of the papers for inclusion. The WHO Malaria Elimination Guideline Development Group considered the findings of these systematic reviews to develop and update recommendations for malaria elimination strategies, published in June 2022.^1^

The first two papers in this supplement and each of the nine intervention-specific reviews and meta-analyses describe a set of studies of specific malaria control measures, including their context, the questions addressed, the methodology for inclusion or exclusion of studies, and the process of assessment of included studies.^1^^,^^2^ The authors explain the challenges of this process and highlight the paucity of rigorous and quality studies for inclusion.

No matter how productive the process of producing multiple reviews and meta-analyses was, the disparity between the breadth of the published literature and the relatively few studies that fulfilled criteria for the evidence base to guide recommendations was remarkable. This creates room for reflection on the quality requirements of information for such analyses. The major reasons for non-inclusion of studies were study design weaknesses, especially due to inadequate study comparison groups or study size. In the realm of implementation science and pilot studies, important questions related to delivery can be answered, but often not in a manner that meets the stringent efficacy and safety requirements that inform policy decisions.

In considering the data and conclusions, the malaria context of countries relative to the recommendations must be clear. The authors highlight that much progress has been made in the past two decades, with 46 countries achieving a level of fewer than 10,000 cases of malaria per year and 23 countries (17 with WHO certification) eliminating their domestic malaria transmission since 2000.^2^^–^^4^ This presumably leaves us with 23 countries "on the verge" of elimination and another 23 countries with recent elimination with the need to now focus on preventing re-establishment of local transmission. These countries are likely the primary audience for these guidance recommendations.

The authors note that the WHO Framework for Malaria Elimination emphasizes that every country, including those with a high malaria burden, should assure that their focus includes approaches that can lead toward reduced transmission and contribute to ultimate elimination.^5^ There are an additional approximate 60 countries with persistent higher endemic malaria transmission. Forty of these countries are in sub-Saharan Africa, accounting for approximately 95% of global cases and deaths.^6^ These 60 countries certainly need to further reduce malaria transmission on the route to elimination, and may have areas of lower transmission where these guidelines have particular relevance.

On the other hand, many of the studies considered in these reviews were in settings with relatively higher transmission seeking to achieve lower transmission, but are still far from the “final phase of elimination”. That is understandable, as often the studies could not easily be done in areas with very low transmission. We note that the use of some of our terminology can create confusion and the words “mass, population-wide, reactive, and targeted” can have different interpretations in higher transmission settings versus places in the final phase of elimination. For example, if transmission is truly very low and focal (e.g., a few cases per year in a district where reaching the case, immediate household, and neighbors and conducting a focus investigation is feasible), reacting to information and targeting this very focused effort may be highly relevant to elimination.

The guidance emanating from these reviews needs continued scrutiny for its relevance to near-elimination and prevention of re-establishment settings.

If we focus on the needs of the 46 countries at or near malaria elimination, these national malaria control programs have already demonstrated strong human capacity, relevant financial resources, and timely information to direct their work. We also have critical information that comes with the WHO requirements for malaria elimination certification.^6^ These requirements provide substantial guidance relevant to case detection, clearance of transmission foci, and documentation of quality surveillance systems that inform elimination. Any additional support to the countries very close to elimination requires flexibility that meets local challenges.

The systematic reviews and meta-analyses described in this supplement and the use of this information for WHO guidance has been a robust effort. A next critical step in this rigorous process is to move beyond global guidance to fully engage and learn from the 46 elimination countries where flexibility in local strategies can facilitate progress to eliminate and ensure no re-establishment of transmission. In this vein, we may be asking not “is this intervention good or not good for eliminating or preventing re-establishment”, but rather “what can this tool or strategy accomplish and what are its limitations?” and “is it right for our local situation now and for how long?”. If similar tools become available, but with better features (e.g., a safe drug with a longer duration of effect; a single dose treatment to replace a multi-day/multi-dose treatment; and a more sensitive diagnostic test for point-of-care), can these programs benefit from past studies that help predict new efficacy opportunities?

Not every study intends to achieve translation into policy recommendations. But, if improving health is the goal, it is critical that we use our scarce research funds to implement well-designed studies and that we use all quality information that is available for program action. The WHO has stressed the importance of a strong research effort to support the Global Malaria Program in its technical strategy and recommendations, but the funding target for research and development has not been achieved. The reviews and meta-analyses contained in this supplement were well considered to inform WHO guidelines. We may now further reflect on how to engage the specific countries and programs that can act on the guidance so that we, as a community, optimize our future questions and relevant designs to yield strategic, targeted, and robust studies that best contribute to improving the fight against malaria.
